# Nerve growth factor-induced Akt/mTOR activation protects the ischemic heart via restoring autophagic flux and attenuating ubiquitinated protein accumulation

**DOI:** 10.18632/oncotarget.14284

**Published:** 2016-12-27

**Authors:** Zhou-Guang Wang, Hao Li, Yan Huang, Rui Li, Xiao-Fan Wang, Li-Xia Yu, Xue-Qiang Guang, Lei Li, Hong-Yu Zhang, Ying-Zheng Zhao, Chunxiang Zhang, Xiao-Kun Li, Rong-Zhou Wu, Mao-Ping Chu, Jian Xiao

**Affiliations:** ^1^ Institute of Cardiovascular Development and Translational Medicine, Children's Heart Center, The Second Affiliated Hospital, Wenzhou Medical University, Wenzhou 325027, China; ^2^ Molecular Pharmacology Research Center, School of Pharmacy, Key Laboratory of Biotechnology and Pharmaceutical Engineering, Wenzhou Medical University, Wenzhou 325035, China; ^3^ Department of Biochemistry and Molecular Biology, College of Basic Medical Science, Jilin University, Changchun, 130012, China

**Keywords:** NGF, myocardial ischemia/reperfusion, autophagy, PI3K/Akt/mTOR, ubiquitin

## Abstract

The dysregulation of autophagy is related to a variety of cardiovascular diseases, such as myocardial ischemia/reperfusion (I/R). Nerve growth factor (NGF) has been shown to have therapeutic potential in ischaemic heart injury. In this study, we demonstrate that NGF administration can accelerate autophagic flux and attenuate protein ubiquitination in myocardial I/R heart. Our results showed that NGF could restored heart function and decreased the apoptosis of cardiomyocytes which induced by myocardial I/R injury. The protective effect of NGF is associated with the inhibition of autophagy related proteins. On another hand, NGF enhances the clearance of ubiquitinated protein and increases the survival of myocardial cell *in vivo* and *in vitro*. Additionally, NGF could activate the PI3K/AKT and mTOR signaling pathways. These results suggested that the cardioprotective effect of NGF is related to the restoration of autophagic flux and attenuation of protein ubiquitination via the activation of PI3K/AKT and mTOR pathway.

## INTRODUCTION

Acute myocardial infarction (AMI) is one of the leading causes of morbidity and mortality worldwide, which has imposed a substantial burden on the society [1]. Several effective reperfusion therapeutic interventions, including coronary artery bypass grafting, thrombolytic therapy and percutaneous coronary intervention, are developed to treat AMI patients [2]. Clinical outcomes are encouraging as these patients exhibited attenuated myocardial infarction, reduced cardiomyocyte apoptosis and restored contractile dysfunction. Despite the success of these reperfusion therapies, there are several shortcomings with this type of intervention including myocardial ischemia reperfusion injury (MIRI) [3, 4]. Over decades, our understanding of the underlying mechanisms of myocardial ischemia reperfusion injury has grown significantly. The pathogenesis reflects the confluence of multiple aspects, including perturbation of inflammatory responses, ischemia, local edema, focal hemorrhage, free radical stress and ion homeostasis. Despite these important progress, there is still a critical lack of successful solutions for prevent MIRI. Our previous studies using both *in vivo* and *in vitro* approaches showed that autophagy is a key player mediating progressive degeneration of the heart, which eventually contributes to the development of myocardial I/R injury [5]. These studies suggest that autophagy could be an effective drug gable target to improve myocardial I/R injury.

Autophagy is an evolutionarily conserved process in response to stress. It is an intracellular degradation system that plays a wide variety of physiological roles in our body's ability to maintain cellular homoeostasis [6, 7, 8]. Dysregulation of autophagy is associated with multiple disorders including cancer, neurodegenerative and cardiovascular diseases [9–11], but whether autophagy is involved in myocardial I/R is still incompletely understood, although some studies have demonstrated that increased autophagic activity is implicated in cell death in the pathogenesis of heart disease [12, 13, 14]. Autophagy is executed by autophagy-related genes (*Atg*), which are conserved from yeast to humans. Among these *Atg* genes, ATG7 encodes the E1 enzyme in the autophagy system and plays a critical role in membrane elongation. ATG7 is not only an important marker of autophagy, but also a critical component regulates cell death and survival [15]. Therefore, previous studies have showed that ATG7 is also implicated in cancer, cardiovascular and neurological diseases [16–19]. However, the role of ATG7 and its associated pathological signaling mechanism in myocardial I/R injury remains to be elucidated.

Nerve growth factor (NGF) is primarily synthesized and secreted by both immature and mature cardiac myocytes. NGF is a neurotrophic factor involved in the regulation of growth and survival of cardiomyocytes [20]. Previous studies have shown that other neuropeptides belonged to the same family of neurotrophins exhibit a cardioprotective effect against myocardial I/R injury [21]. Overexpression of NGF and its high-affinity receptor, tyrosine kinase (TrkA), in the ischemic rat and human hearts promote cellular survival in ischemic myocardium [22, 23]. The cardioprotective effect of NGF might be associated with the activation of its downstream phosphatidylinositol 3-kinase (PI3K) signaling pathway [24].

In this study, we sought to examine whether NGF improves cardiomyocyte survival and promotes functional recovery against myocardial I/R injury. We found that treatment of NGF inhibited autophagic activity by the activation of its downstream PI3K/Akt/mTOR signaling following myocardial I/R in mice. Collectively, our results suggest that NGF is potential therapeutic approach for treating the ischemic heart in humans.

## RESULTS

### NGF improves cardiac function in a mouse model of myocardial I/R injury

To examine the cardioprotective effect of NGF on cardiac function after myocardial I/R injury in mice, echocardiographic was used in our study to test cardiac contractility. After 3d of reperfusion, increased average LVEDd (3.7 ± 0.3 mm) and LVESd (2.3 ± 0.2 mm) were observed in the I/R group (Table 1), and these readings were significantly higher than the recordings in the control group suggesting that myocardial I/R led to ventricular dilation in mice. Interestingly, the I/R group received NGF treatment showed lower average LVEDd value of 3.3 ± 0.1 mm and LVESd value of 1.8 ± 0.1 mm when compared to the non-treated I/R group. The left EF was decreased (81.0 ± 3.3 %) in the control group as relative to the myocardial I/R animal model group (57.9 ± 2.5 %). After NGF treatment, the EF was reversed to 67.1 ± 3.9 % in the myocardial I/R animal model group, which was consistent to the FS results showing improved cardiac function. In summary, these results suggested that treatment of NGF effectively ameliorated cardiac function after myocardial I/R injury in mice.

**Table 1:** Echocardiographic assessment showed that NGF could improve cardiac function

Parameters	Sham	I/R	I/R+NGF
LVEDd	3.0±0.2	3.7±0.3*	3.3±0.1^#^
LVESd	1.4±0.2	2.3±0.2*	1.8±0.1^#^
EF(%)	81.0±3.3	57.9±2.5**	67.1±3.9^#^
FS(%)	56.4±3.1	35.4±1.8**	43.5±2.3^#^

LVESd: left ventricular end systolic dimension. LVEDd: left ventricular end diastolic dimension. EF: ejection fraction. FS: fractional shortening. * represents *P* < 0.05 vs. Sham group. ** represents *P* < 0.01 vs. Sham group. ^#^ represents *P*<0.05 vs. I/R group, Values are mean ± SD.

### NGF decreases myocardial apoptosis and fibrosis in a mouse model of myocardial I/R injury

**Figure 1:**
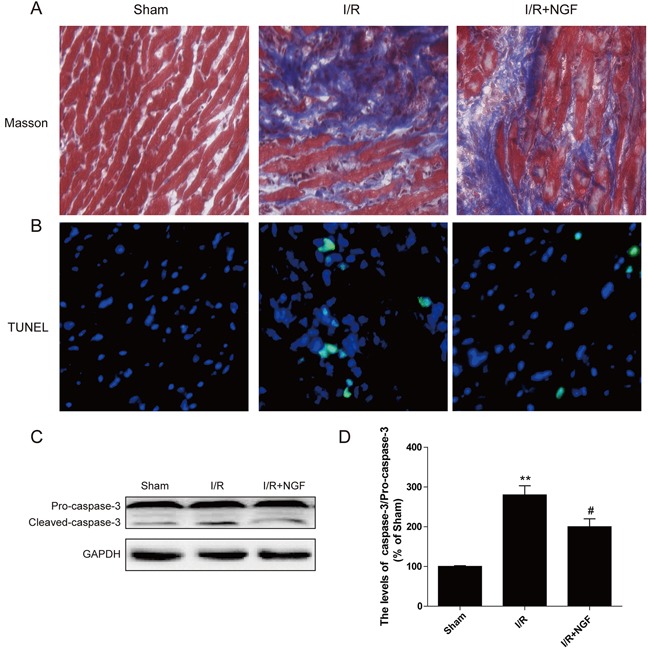
NGF attenuated myocardial apoptosis and fibrosis in myocardial I/R heart **A**. The Masson's trichrome staining results showed the fibrosis in the border zone at 3 days after injury. **B**. TUNEL immunofluorescent staining of sections from the injury area in myocardial I/R heart. **C**. The protein expression of cleaved-caspase-3 in myocardial I/R heart. **D**. The optical density analysis of cleaved-caspase-3 expression in theI/R heart. * *P* < 0.05, ** *P* < 0.01, versus the sham group,^#^ represents *P* < 0.05, ^##^ *P* < 0.01 versus the I/R group. The mean values ± SEM, n = 6 per group. I/R, ischemia/reperfusion.

No cell death was observed as absence of TUNEL-positive apoptosis was found in the control group (Figure 1A). A significant increased amount of TUNEL-positive cells was found in mice after I/R for 3 d. However, treatment of NGF markedly attenuated the number of myocardial apoptotic cells (Figure 1A). Increased expression of cleaved caspase-3 protein was found in the mouse hearts after myocardial I/R, which was significantly reversed by treatment of NGF (Figure 1C and 1D). Myocardial ischemia presents with obvious myocardial necrosis and fibrosis. Our Masson staining showed that myocardial I/R injury resulted in a significant amount of collagen deposition in the infarct area in mice, and treatment of NGF effectively reduced the collagen content in the border zone (Figure 1B). Absence of collagen deposition was observed in the control group. Taken together, these data indicated that NGF protected the myocardium by reducing apoptosis and fibrosis in the infarcted heart.

### The cardioprotective effect of NGF is related to restoration of autophagic flux in a mouse model of myocardial I/R injury

**Figure 2:**
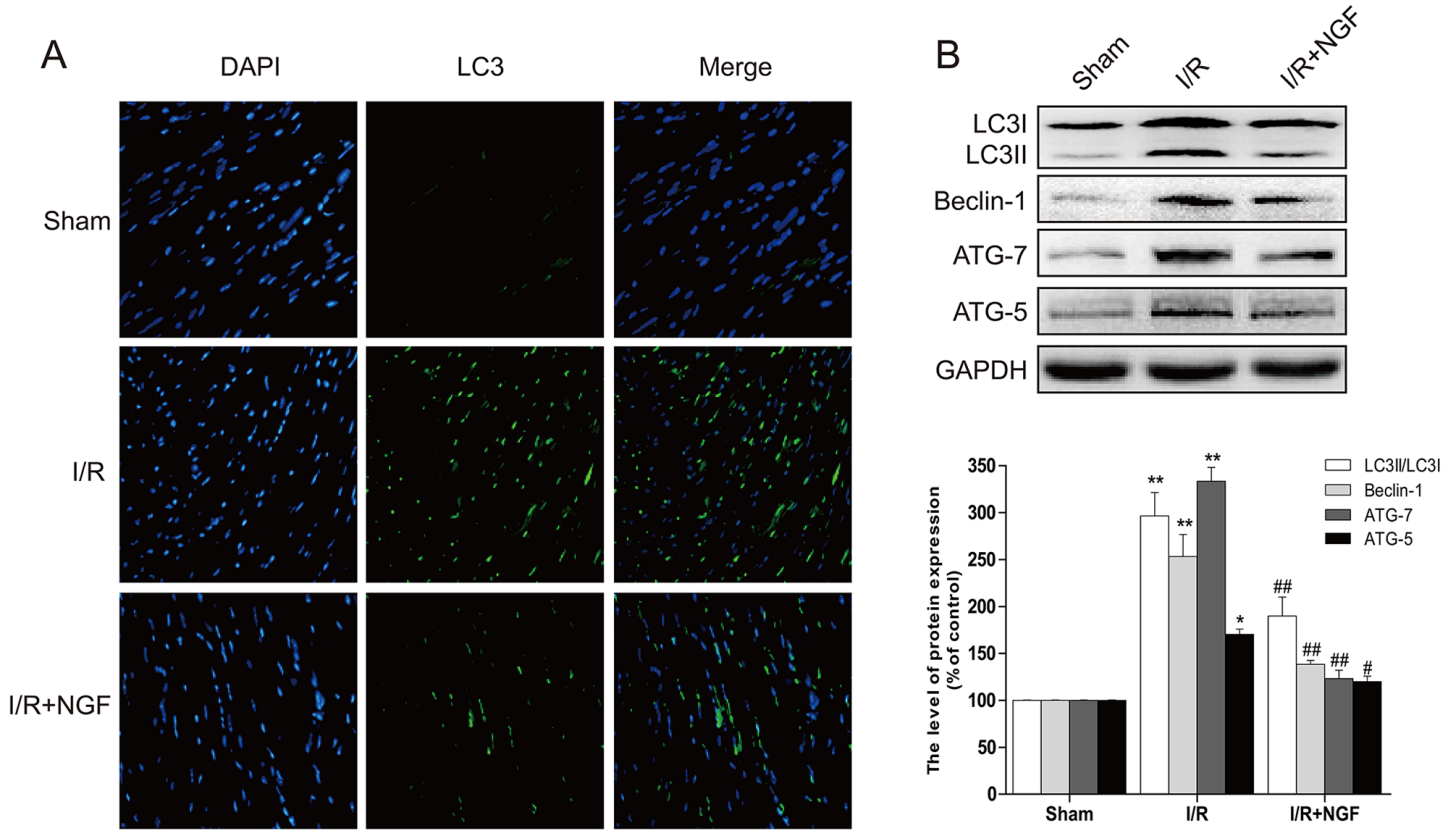
NGF attenuated the autophagy related proteins expression in the injury area of myocardial I/R heart **A**. Representative immunofluorescent staining results of LC3 (marked as green) in ischemic area of the mice hearts, the nucleimarked as blue with hoechst. **B**. The protein expression of autophagy related proteins in the injury area of myocardial I/R heart. **P < 0.01 versus the sham group, ^#^*P* < 0.05 versus the I/R group. The mean values ± SEM, n = 6.

We sought to study the underlying mechanism by which NGF protects myocardium, and examined whether this protective effect was associated with inhibition of autophagic activities. The mammalian autophagy protein, LC3, is a marker of autophagosomes. Our double immunofluorescence staining showed that the amount of LC3-positive cells significantly increased at the heart lesion site when compared to the sham group, but the number of LC3-positive cells was down regulated in NGF-treated mice with myocardial I/R injury (Figure 2A). Western blot analysis also demonstrated that the ratio of LC3II/LC3I and the level of other autophagy-related proteins, including beclin-1, ATG-5 and ATG-7, significantly increased in the non-treated myocardial I/R injured group, but up-regulation of these autophagy markers were markedly reversed by treatment of NGF (Figure 2B). Previous studies have shown that Chloroquine (CQ) could inhibit the clearance of autophagosomes by interfering with the fusion of autophagosomes with lysosomes, and therefore reduces lysosomal degradation. CQ also increases the LC3II protein expression in normal mouse heart, but it does not significantly affect the level of LC3II protein expression in the heart with impaired autophagic activity. Thus, the level of LC3II protein expression after CQ treatment could be used to assess whether autophagosome clearance is disrupted. In the absence of CQ, the expression of LC3II protein in the myocardial I/R injured mice was markedly increased when compared with the control group (*P* < 0.05). The expression of LC3II protein was increased in the control group received treatment of CQ (*P* < 0.01). However, no significant differences in the level of LC3II protein were observed between themyocardial I/R group received treatment of CQ treatment and the non-treated myocardial I/R group (*P* > 0.05) (supplemental Figure 1E-1F). There were no significant differences found between the CQ-treated myocardial I/R group and non-treated myocardial I/R group on heart function as revealed the echocardiographic analyses (supplemental Figure 1A-1D). In summary, treatment of NGF was able to accelerate the autophagic flux by enhancing the degradation of autolysosomes, and decreased the level of LC3II protein expression (supplemental Figure 1G-1H).

### NGF improves functional recovery in the heart via enhancing autophagic clearance of ubiquitinated protein accumulation in a mouse of model of myocardial I/R injury

**Figure 3:**
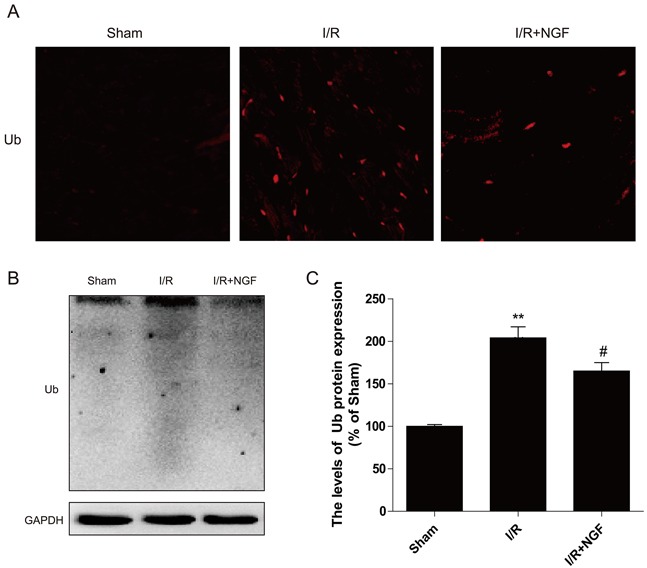
NGF clears ubiquitinated proteins accumulation after myocardial I/R **A**. Representative immunofluorescent staining results of Ub (red dot) and DAPI (blue dot) in ischemic area of the mice hearts. **B**. The protein expression of Ub in ischemic area of the mice hearts. **C**. The optical density image analysis of Ub protein. **P < 0.01 versus the sham group, ^#^ represents *P* < 0.05 versus the I/R group. The mean values ± SEM, n = 6.

Polyubiquitin chains regulate a variety of biological processes, including an initiation of proteolysis of substrates. To determine whether treatment of NGF impacts protein degradation and homeostasis, our western blot analyses showed that poly-ubiquitinated protein levels were markedly increased in mouse hearts after myocardial I/R injury (Figure 3B), and these findings were further confirmed by double immunofluorescence staining showing that significantly increased poly-ubiquitinated protein expression at the heart lesion site. Additionally, treatment of NGF decreased the accumulation of poly-ubiquitinated protein (Figure 3A), suggesting that NGF promoted recovery after reperfusion injury via enhancing the autophagic protein degradation.

### The PI3K/Akt/mTOR signaling pathway is involved in the cardioprotective role of NGF in a mouse model of myocardial I/R injury

**Figure 4:**
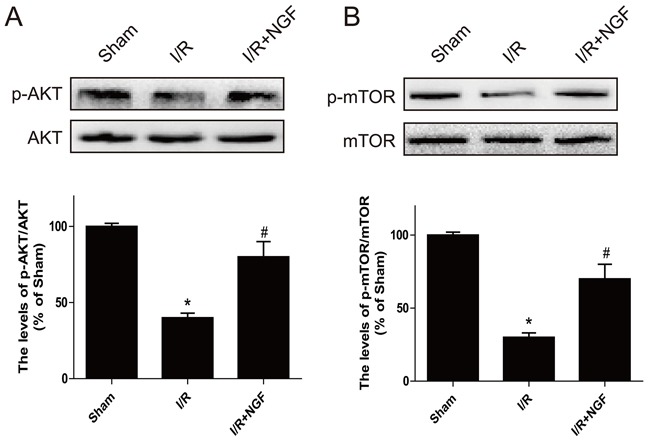
The PI3K/AKT and mTOR signaling pathways are activated by NGF treatment three days after ischemia/reperfusion injury **A**. Western blot results showed the expression of p-mTOR (Ser2448)/mTOR, p-Akt (Ser473)/Akt in ischemic area of the mice hearts **B**. The optical density image analysis of the p-mTOR (Ser2448)/mTOR and p-Akt(Ser473)/Akt proteins. The mean values ± SEM, n = 6, **P < 0.01 versus the sham group, ^#^ *P* < 0.05 versus the I/R group.

The PI3K/Akt signaling pathway is essential for cardiomyocyte proliferation and differentiation. The PI3K/Akt signaling directly regulates its downstream effect or mTOR, which inhibits autophagy. Our western blot analysis demonstrated that activation of the Akt/mTOR pathway was significantly decreased after myocardial I/R injury in mice (Figure 4A and 4B), whereas treatment of NGF markedly enhanced the PI3K/Akt pathway in the myocardial I/R treatment group (*P* < 0.05). These data suggested that the cardioprotective role of NGF was in part by activation of the PI3K/Akt/mTOR pathway after myocardial I/R injury in mice.

### NGF protects H9C2 cells and HUVECs through restoring the autophagic flux *in vitro*

**Figure 5:**
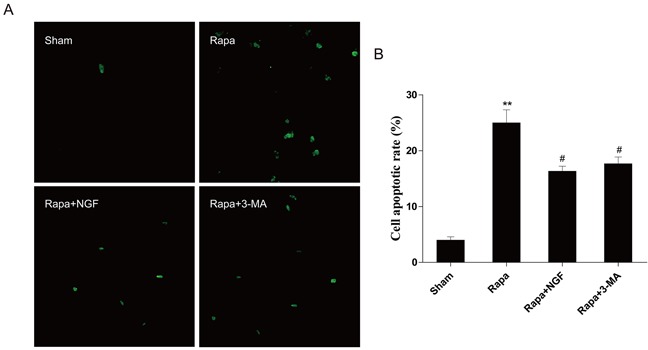
NGF protected H9C2 cells from rapamycin-induced apoptosis **A**. H9C2 cells were incubated with rapamycin (100 ng/ml) with or without NGF (50 ng/ml). The apoptotic cell were detected by TUNEL staining. The green dot indicates the apoptotic cell. **B**. The analysis of the apoptotic cell rate. **P < 0.01 vs. the Sham; ^#^*P* < 0.05 vs. the rapamycin group.

**Figure 6:**
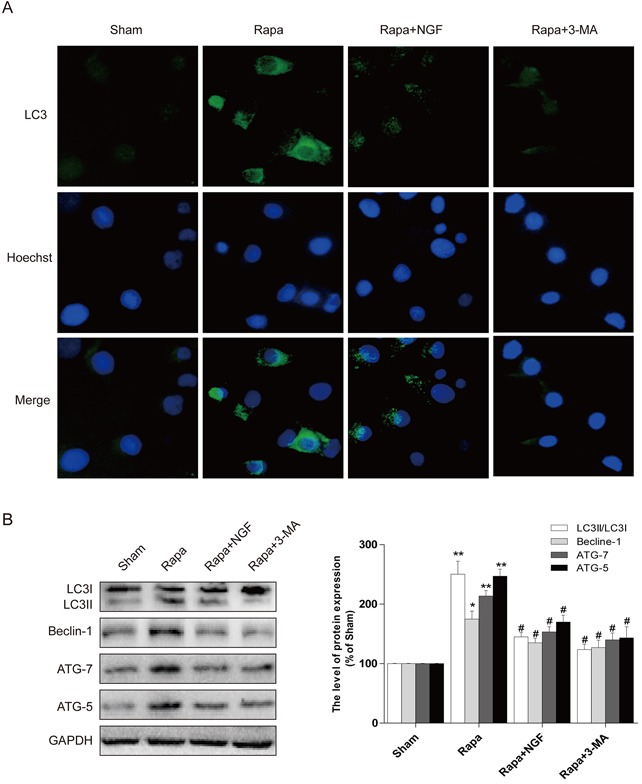
NGF protected H9C2 cells from rapamycin-induced apoptosis through inhibiting autophagy **A**. Representative immunofluorescent staining results of LC3 (marked as green) in H9C2 cells, the nuclei marked as blue with hoechst. **B, C**. the autophagy related proteins expression in H9C2 cells. The optical density image analysis of autophagy related proteins. * *P* < 0.05 versus the sham group, ^#^ *P* < 0.01 versus the rapamycin group.

To confirm our in vivo findings, we further examine whether NGF-mediated autophagic flux protects myoblast survival. H9C2 myoblasts (an alternative cell model for cardiomyocytes) were treated with rapamycin or co-treated with NGF and rapamycin or 3-MA with rapymycin. A significant increase in H9C2 cell apoptosis, as indicated by elevated TUNEL staining, was observed in the rapamycin-treated group. However, treatment of NGF has shown protection against rapamycin-induced cell death in the culture system. In parallel, treatment of 3-MA also reversed rapymycin-induced cell death, suggesting that NGF promoted restoration of autophagic flux was required for H9C2 cell survival when challenged with rapamycin (Figure 5A and 5B). The LC3 puncta accumulation in H9C2 cells was determined by double immunofluorescence staining. H9C2 cells treated with NGF showed significant increase in the LC3 puncta when compared to the group treated with rapamycin alone (Figure 6A), and the LC3 puncta was also increased in the H9C2 cells when treated with 3-MA. These results were further confirmed by western blot analyses showing that increased LC3II, ATG-7, ATG-5 and beclin-1 at the protein levels in rapamycin-treated H9CS cells, which were significantly down-regulated by co-treatment of NGF or 3-MA (Figure 6B and 6C). Collectively, these data indicated that NGF mediated restoration of autophagic flux in vitro. And HUVEC were also treated with rapamycin or combined with NGF, as showed in our results, NGF could also restore autophagic flux in HUVEC (Supplemental Figure 2).

**Figure 7:**
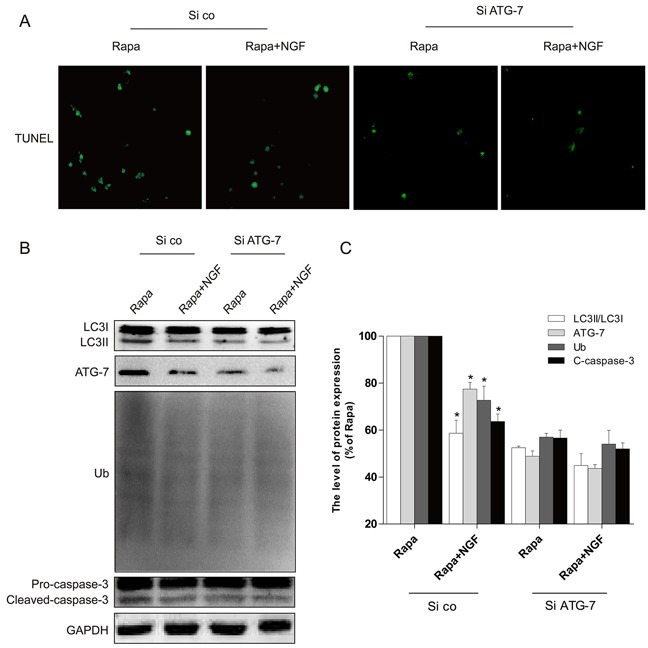
Silencing of ATG-7 partially blocked the harmful effects of rapamycin **A**. H9C2 cells were incubated with rapamycin (100 ng/ml) with or without NGF (50 ng/ml). The apoptotic cell was detected by TUNEL staining. **B, C**. The autophagy related proteins expression in H9C2 cells. *P < 0.01 versus the rapamycin group. Rapa, rapamycin. si ATG-7: ATG-7 siRNA, si co: random siRNA.

**Figure 8:**
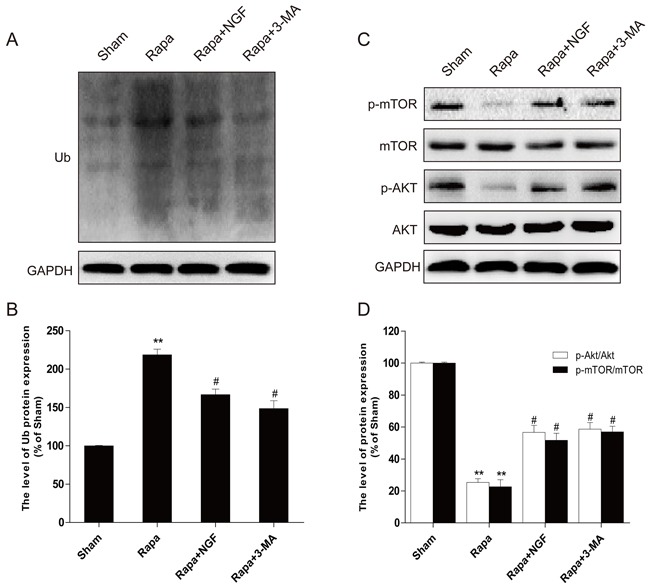
NGF could inhibit rapamycin-induced protein ubiquitination through PI3K/AKT and mTOR pathways in H9C2 cells **A,B**. Western blot image results and optical density image analysis of Ub protein expression in H9C2 cells. **C,D**. Western blot image results and optical density image analysis of p-mTOR (Ser2448) and p-AKT (Ser473) in H9C2 cells. *P < 0.05, **P < 0.01 versus the sham group, ^#^*P* < 0.05 versus the rapamycin group.

*In vitro* siRNA studies on H9C2 cells were used to further examine whether NGF-mediated restoration of autophagic flux is required for the protective effect of NGF against rapamycin-induced apoptosis (Figure 7A). The efficiency of knockdown of ATG-7 mediated by siRNA in H9C2 cells was confirmed by western blot (Figure 7B and 7C). We found that knockdown of ATG-7 significantly reduced the conversion of LC3-I to LC3-II and Ub expression, suggesting autophagy was inhibited (Figure 7B). H9C2 cells with ATG-7 knockdown also showed improved cell survival when challenged with rapamycin, but significant increased cell death was mediated by rapamycin in H9C2 cells treated with control siRNA. Taken together, these results demonstrated that targeted deletion of autophagy gene ATG-7 in H9C2 cells blocked the harmful effects mediated by rapamycin, which was consistent with the hypothesis that cells treated with NGF protected H9C2 cell survival against rapamycin by a mechanism that is involved restoration of autophagic flux. We next investigated how NGF promoted cell survival through enhancing autophagic flux and clearing poly-ubiquitinated protein accumulation *in vitro*. As indicated in Figure 8A, treatment of rapamycin significantly increased poly-ubiquitinated protein expression and that was markedly suppressed by treatment of NGF. The PI3K/Akt/mTOR pathway was up-regulated by NGF in H2C9 cells as compared to the experimental group that was treated with rapamycin alone (Figure 8C and 8D). Taken together, these *in vitro* findings suggested that NGF promoted cardiac cardiomyocyte through the activation of the PI3K/Akt/mTOR signaling pathway.

## DISCUSSION

MIRI typically arises in patients with an acute myocardial infarction. The process of reperfusion induces extensive cardiomyocyte death, which causes myocardial infarction is a major cause of morbidity and mortality. After myocardial ischemia, the process of myocardial reperfusion provokes a wide variety of detrimental effects, including necrosis, apoptosis oxidative stress and inflammation. The loss of functional cardiomyocyte leads to secondary damages that interfere with recovery from myocardial infraction. Previous studies showed that the mechanisms underlying myocardial I/R injury might be associated with calcium overload and mitochondrial dysfunction [25], an increase in reactive oxygen species [26], activation and adhesion of neutrophils [27], or cellular apoptosis [28]. Others have reported that NGF delivered during reperfusion protects the heart from MIRI [23, 29, 30], but the mechanism by which NGF regulates cardiomyocyte survival and function remains to be elucidated. In the present study, we found that NGF improves MIRI in a mouse model, and the protective effect of NGF is associated with increased autophagy-mediated ubiqutintation.

Autophagy is an evolutionarily biological mechanism involved in the degradation of damaged proteins and organelles [31]. A large body of studies have previous suggested that autophagy plays an adaptive role in protecting cardiomyocytes after MIRI, which suppress the process accelerating heart failure [32]. However, in recent years, emerging evidence has revealed that dysregulated autophagy is detrimental. It has been shown that autophagy is associated with a variety of pathological disorders including spinal cord injury, neurodegenerative and cardiovascular diseases. Dysregulated autophagy contributes to cell death after MIRI [33], knockdown of Beclin1 mediated by RNAi approach resulted in enhanced cardiomyocyte survival. Impaired autophagosome clearance and ROS-induced autophagy also contribute to cardiomyocyte death after MIRI. Furthermore, MIRI leads to increased production of oxidative stress that may exaggerate autophagic activity-induced death in cardiomyocytes [34]. Our study has confirmed these findings showing that autophagy-induced apoptosis played a large role in mediating secondary damages after MIRI. Mechanistically, NGF restored autophagic flux by activation of the PI3K/Akt/mTOR pathway promoting cardiomyocyte survival. Despite these findings, the role of autophagy in MIRI is still controversial, as most studies have proposed that autophagy is a protective mechanism in the heart. The present study revealed that CQ suppressed the fusion of autophagosomes with lysosomes, resulting in increased expression of LC3II protein. However, the effect of CQ was diminished after MIRI in the mouse heart, implying that MIRI blocked the clearance of autophagosome, and altered LC3II protein expression during MI might be associated with disrupted autophagic flux and an enhanced autophagic activity. Taken together, this is the first study demonstrating that treatment of NGF restored autophagic flux by enhancing the degradation of autolysosomes, and activation of this pathway is required for cardiomyocyte survival and recovery after MIRI in mice.

The stimulation of NGF activates the PI3K/Akt pathway promotes cellular proliferation, differentiation, and survival. More importantly, Moreover, the PI3K/Akt pathway is essential for cardiomyocyte cell survival under a wide variety of circumstances. Previous studies have shown that the PI3K/Akt pathway phosphorylates its downstream effector, known as mTOR, providing a cardioprotective effect, and this was associated with reduced autophagic activity [35]. Rapamycin has been shown to be able to induce autophagy via mTOR inhibition [36]. Overexpressing mTOR in the heart was able to suppress the inflammatory response providing substantial cardioprotection against MIRI in mice. In the present study, we observed that autophagic flux was impaired during the early stage of MIRI in mice, and treatment of NGF activated the downstream PI3K/Akt/mTOR pathway, which restored autophagic flux that was essential for cardiomyoctye survival and function. These findings were further supported by observations from our in vitro studies. In H9C2 cells, both NGF and 3-MA were able to reverse cell death mediated by rapamycin. Furthermore, knockdown of the Atg7 gene expression significantly attenuated H9C2 cell death induced by rapamycin. Taken together, these findings suggested that the cardioprotective effect of NGF is involved in autophagy inhibition via activation of the Akt/mTOR signaling pathway.

The ubiquitin-proteasome system (UPS) plays multiple roles in several myocardial diseases [37]. The proteasomal and lysosomal degradation were historically regarded as two parallel pathways, but emerging evidences suggest that two pathways actually interact with each other and their interplay is critical to maintaining proteostasis in the cell [38, 39]. Previous studies has demonstrated that proteasome inhibition activated macroautophagy in cell culture and mice [40]. In the present study, we have unveiled the functional role of NGF on UPS function. Impaired UPS leads to misfolded protein aggregation, and excessive ubiquitinated protein accumulation itself might lead to UPS dysfunction. In this study, our results showed that NGF markedly enhances the clearance of ubiquitinated proteins in myocardial I/R heart. Silencing of ATG-7 partially improved the ability to clear ubiquitinated proteins and prolonged the survival of H9C2 cell, indicating that NGF may inhibit excessive autophagic cell death and was able to reverse baneful accumulation of ubiquitinated proteins via the target ATG-7 protein.

In conclusion, treatment of NGF significantly improved cardiomyocyte survival, and recovered the heart damage after MIRI in mice. The protective effect of NGF is related to restoration of authophagic flux and enhanced ubiquitinated protein clearance through inhibition of ATG-7 protein expression. Mechanistically, NGF mediated activation of the PI3K/Akt/mTOR which is required for myocardial cell survival. Our study suggests that NGF may be a suitable candidate for new therapeutic treatment for treating MIRI.

## MATERIALS AND METHODS

### Animal

Male C57/B6 mice at 2 to 3 monthes old (Animal Center of the Chinese Academy of Sciences) were used in this study. All animal work was performed under protocols approved by the Institutional Animal Care and Use Committee at Wenzhou Medical University.

### Antibodies and reagents

NGF was purchased from Sigma (Sigma-Aldrich, St.Louis, MO). Fetal bovine serum (FBS), Dulbecco's modified Eagle's medium (DMEM) and other cell culture media were purchased from Invitrogen (Invitrogen, Carlsbad, CA). Primary antibodies against total Akt, phospho-Akt (Ser473), total mTOR, phospho-mTOR, cleaved-caspase-3, LC3, ATG-7, ATG-5, Beclin-1 and GAPDH were purchased from Santa Cruz Biotechnology (Santa Cruz, CA, USA). Goat antibodies anti-rabbit and anti-mouse IgG-HRP were purchased from Cell Signaling Technology, Inc. (Danvers, MA, USA). Rapamycin and 3-Methyladenine were purchased from Sigma (Sigma-Aldrich, St. Louis, MO).

### Cell culture and viability assay

Human umbilical vein endothelial cells (HUVECs) were purchased from ATCC, Manassas, VA. Rat cardiomyocyte H9C2 cells were purchased from the American Type Culture Collection. Cells were cultured in DMEM supplemented with 10% FBS, 5% horse serum, and antibiotics (100 units/ml penicillin, 100 μg/ml streptomycin). All cells were cultured in a humidified atmosphere containing 5% CO_2_ at 37°C. Based on our previous study, cells were plated in 6-well plates or 35-mm culture dishes at 5.0×10^4^ cells/cm^2^ and then incubated in 100 nM rapamycin, NGF (50 ng/ml), NGF with rapamycin or 3-methyladenine (3-MA, 5 mM) with rapamycin.

### Mice myocardial I/R model

The induction of mice myocardial I/R model was performed as previously described [5]. Mice received operation were assigned randomly with n = 6 to 15 in each experimental group. Animals received the same surgical procedure without the coronary artery ligation was assigned to the control group. For the treatment groups, mice with myocardial I/R injury were treated with 2 μg NGF each mouse (intravenous treatment) or 10 mg/kg chloroquine (intraperitoneal treatment) at before reperfusion.

### Echocardiography

Echocardiographic studies were performed by a blinded investigator repeatedly before surgery and at 3 days post-surgery to assess the cardiac function as we previously described [5]. Echocardiographic parameters were measured using a high-frequency linear probe (12 MHz phased-array SONOS-7500 transducer, PHILIPS, the Netherland). Functional parameters, including the LV fractional shortening (LVFS), LV fractional area change (LVFAC), and LV ejection fraction (LVEF), were determined as previously described [5]. Mice which died, displayed behavioral abnormality, or were sacrificed for histological analysis were not included in echo cardiographic studies.

### TUNEL assay

Apoptosis in animal hearts was determined by a TUNEL Apoptosis Assay KIT (Roche, Mannheim, Germany) according to the manufacturer protocol [41]. The images were captured with a Nikon ECLIPSE Ti microscope (Nikon, Japan). The H9C2 cells apoptosis rate was measured by a PI/Annexin V-FITC kit (Invitrogen, Carlsbad, CA, USA) and then analyzed by a FACScan flow cytometer (Becton Dickinson, Franklin Lakes, NJ, USA) as we previously described [5].

### Masson staining

Animal hearts were harvested at 3 days post-infarction. Hearts were either fixed in 10% formalin and embedded in paraffin or prepared for cryosections. Hearts were serially sectioned (8 mm thick) from apex to the ligation positions we previously described [5]. Collagen fibers were identified by Masson's trichrome kit according to the manufacturer protocol (IMEB, San Marcos, CA). Quantitative evaluation for collagen deposition was analysis by Image J. A minimum of 12 sections from each group was analyzed.

### Immunofluorescence staining

To evaluated the LC3 and Ub proteins expression, non-specific antibody binding was blocked with 10% donkey or goat serum for 1-2 hours at room temperature (RT). Sections were stained with primary antibodies of appropriate dilution at 4°C overnight (LC3 (1:200) or cleaved Ub (1:200)). Fluorescent secondary antibodies were obtained from Invitrogen, and immunofluorescent images were taken by Nikon ECLPSE 80i fluorescence microscope equipped with a QImaging Retiga 1300 camera [42, 43].

### Western blot analysis

Total proteins from animal hearts or H9C2 cells were extracted using protein extraction reagents. Equal amounts of protein were separated by 12% gel and then transferred to a PVDF membrane. After blocking with 5% bovine serum albumin, membranes were incubated with primary antibodies with shaking at 90 rpm overnight. Membranes were washed with TBS buffer and treated with secondary antibodies for 2 h at room temperature. The signals were visualized with the ChemiDic XRS+ Imaging System (Bio-Rad Laboratories, Hercules, CA, USA). The band densities were quantified with Image J (National Institutes of Health) [44, 45].

### ATG-7 small interfering RNA (siRNA) transfection

H9C2 cells (5×10^5^ cells/well) were cultured in 6-well plates and treated for 4-6 h with control siRNA or ATG-7 siRNA using Opti-MEM media (Gibco Life Technologies, Inc) according to a siRNA transfection protocol described by the manufacturer. Cultured media was replaced by antibiotic-free DMEM after the transfection, and H9CS were maintained in fresh DMEM for 24 h before harvesting for subsequent experiments.

### Statistical analysis

Data are expressed as the mean ± SEM. Statistical significance was determined by Student *t*-test if comparing only two groups or one-way ANOVA followed by Dunnett's post-*hoc* test if analyzing more than two groups. Differences were considered to be statistically significant when *P* values < 0.05.

## SUPPLEMENTARY FIGURES


