# Supplementary material for: Nerve growth factor-induced Akt/mTOR activation protects the ischemic heart via restoring autophagic flux and attenuating ubiquitinated protein accumulation
Source: Oncotarget. 2016 Dec 27;8(3):5400–13. doi: 10.18632/oncotarget.14284 (PMC5354918; doi:10.18632/oncotarget.14284)
Supplement: Supplementary file 1 [file oncotarget-08-5400-s001.pdf]

# Nerve growth factor-induced Akt/mTOR activation protects the ischemic heart via restoring autophagic flux and attenuating ubiquitinated protein accumulation

## SUPPLEMENTARY FIGURES

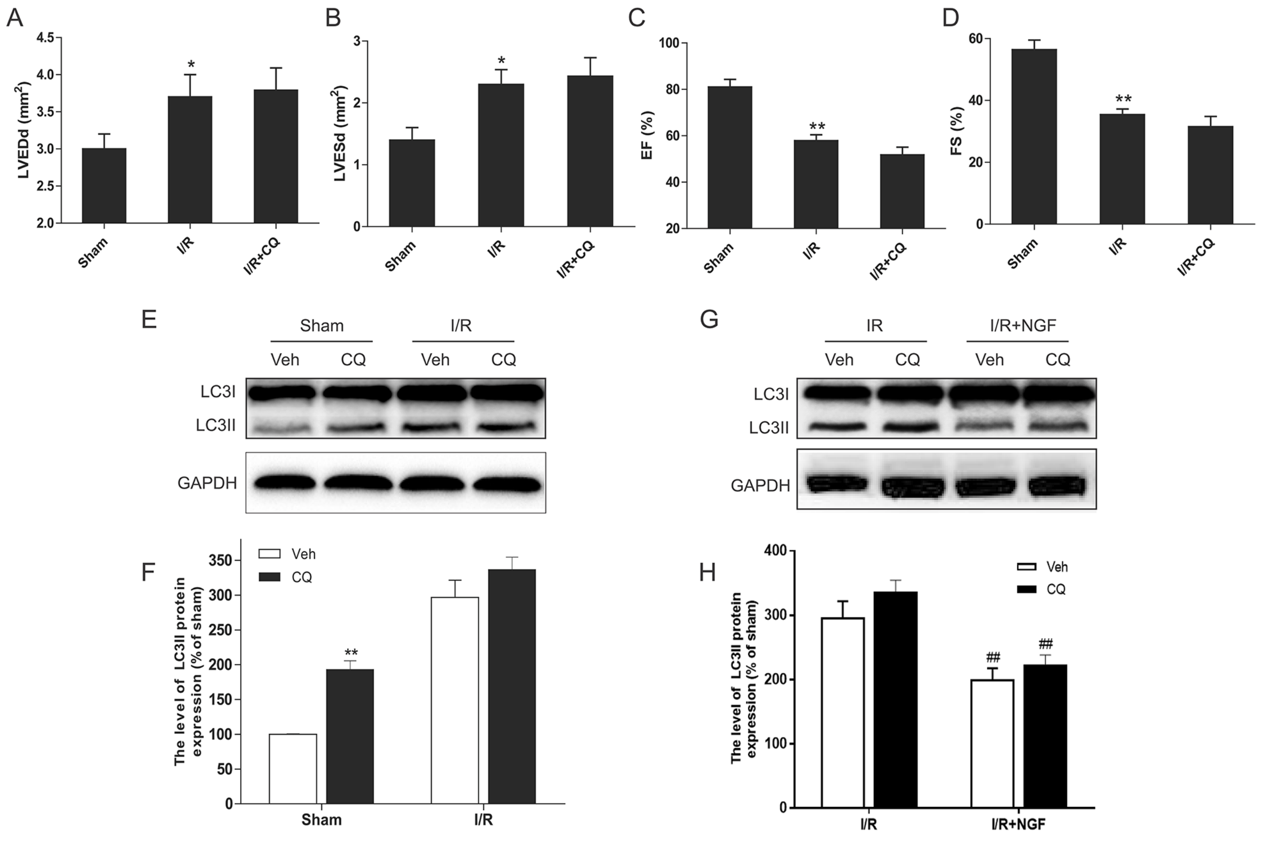


**Supplementary Figure S1: Echocardiographic assessment and change of LC3II in sham, MIRI groups and CQ treatment groups.** **A.** Left ventricular end diastolic dimension results of sham, MI/R and CQ treatment groups. **B.** Left ventricular end systolic dimension results. **C.** Left ventricular ejection fraction results. **D.** Left ventricular fractional shortening results. **E-F.** Change of LC3II in sham, and MI/R groups. **G-H.** Change of LC3II in sham, and MI/R groups. *$P < 0.05$, **$P < 0.01$ versus the sham group.

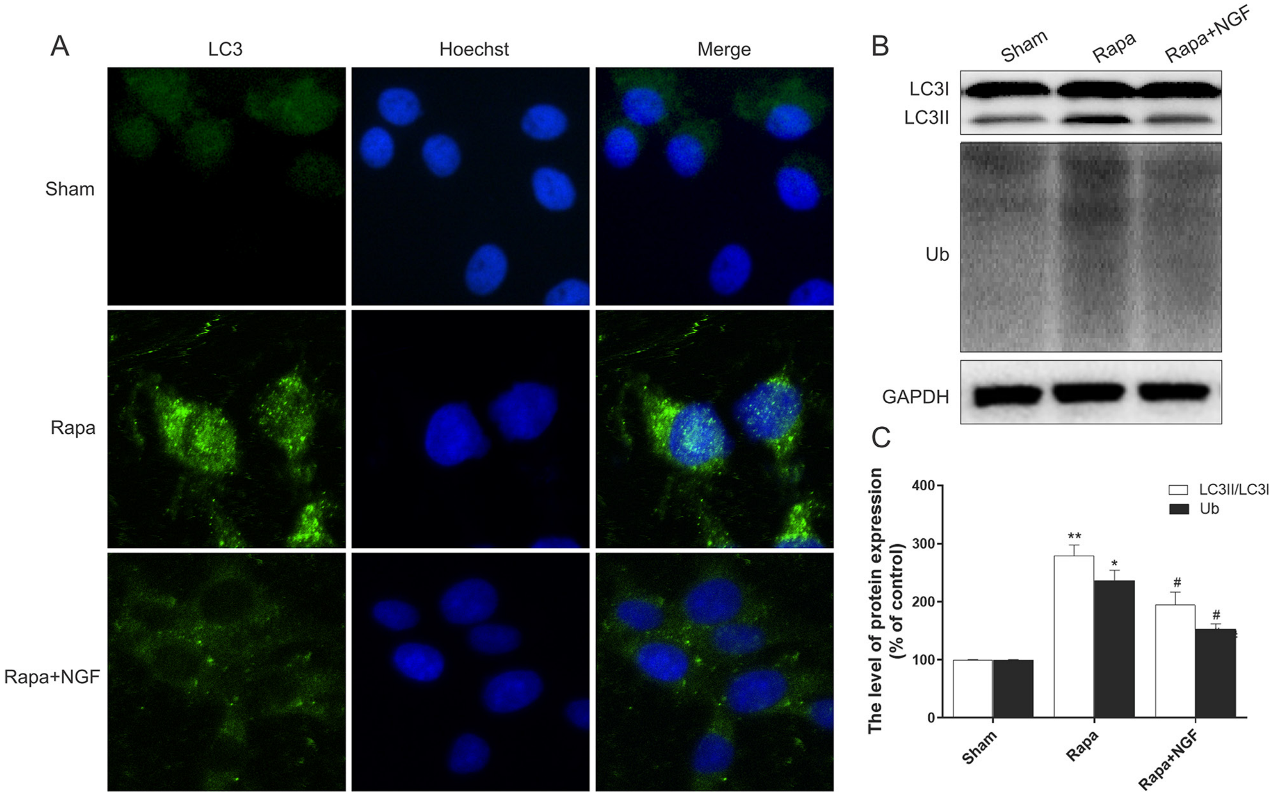


**Supplementary Figure S2: The effects of NGF on rapamycin-induced by autophagy in HUVEC. A.** Representative immunofluorescent staining results of LC3 (marked as green) in HUVEC cells, the nucleimarked as blue with hoechst. **B, C.** The autophagy related proteins expression in HUVEC cells. The optical density analysis of LC3II/LC3I, Ub in the sham, rapa group, and rapa treated with NGF. * $P < 0.05$ ** $P < 0.01$ vs sham group, $^{\#}$ $P < 0.01$ vs rapamycin group.
